# Prospective study of high-dose chemotherapy and autologous peripheral stem cell transplantation in adult patients with advanced desmoplastic small round-cell tumour

**DOI:** 10.1038/sj.bjc.6601304

**Published:** 2003-09-30

**Authors:** A Bertuzzi, L Castagna, V Quagliuolo, V Ginanni, S Compasso, M Magagnoli, M Balzarotti, A Nozza, L Siracusano, I Timofeeva, B Sarina, H Soto Parra, A Santoro

**Affiliations:** 1 Department of Medical Oncology and Hematology, Istituto Clinico Humanitas, Via Manzoni 56, Rozzano, Milan 20089, Italy; 2 Department of General Surgery, Istituto Clinico Humanitas, Rozzano, Milan, Italy

**Keywords:** desmoplastic small round-cell tumour, high-dose chemotherapy

## Abstract

A total of 10 desmoplastic small round-cell tumour patients were treated by high-dose chemotherapy with stem cell support. After high-dose chemotherapy, no complete response conversion was obtained and EWS-WT1 fusion transcript detection was positive in the peripheral blood during follow-up in all patients. High-dose chemotherapy did not seem to change the results in desmoplastic small round-cell tumour.

Desmoplastic small round-cell tumour (DSRCT) is a rare and aggressive disease affecting mainly young male patients. Large masses in the abdomen with compressive symptoms are frequently observed. Morphological, immunohistochemical, and cytological characteristics have been previously described (Gerald *et al*, 1998). A recurrent chromosomal translocation t(11;22)(p13;q12) has been found in tumour cells with detection of a chimeric transcript EWS-WT1 using PCR.

Treatment of DSRCT is a challenge because radical surgery is infrequently performed, and conventional chemotherapy induces short-lived responses (Farhat *et al*, 1996).

Recently, the Memorial Sloan-Kettering Cancer Center group reported interesting results using an intensive alkylator-based protocol associated to surgery and radiotherapy (Kushner *et al*, 1996).

Previously, we reported the results of high-dose chemotherapy in patients with small round-cell tumour (Bertuzzi *et al*, 2002). Seven out of 28 of them were affected by DSRCT.

Here, we report the clinical and molecular results obtained in 10 adult DSRCT prospectively treated by high-dose chemotherapy and autologous peripheral stem cell transplantation.

## PATIENTS AND METHODS

### Eligibility criteria

Patients with DSRCT aged 15–60 years were considered eligible for the study. Disease staging was determined using chest X-rays, technetium 99m bone scan, computed tomography or magnetic resonance imaging, and the evaluation of bone marrow specimens. The diagnosis was established on the basis of histochemical findings as previously described (Gerald *et al*, 1998).

Informed consent for the treatment was obtained in accordance with our Institutional Review-Board guidelines.

### Treatment protocol

The treatment programme combined conventional chemotherapy, local treatment (surgery and/or radiotherapy), and myeloablative therapy with autologous stem-cell rescue. The chemotherapeutic programme consisted of a four-course induction phase with epirubicin 30 mg m^−2^, days 1–3; ifosfamide 3 g m^−2^, days 1–3; and vincristine 2 mg, day 1 (IVE), every 3 weeks, followed by a mobilisation phase with high-dose etoposide (2.4 g m^−2^) or cyclophosphamide (7 g m^−2^), with G-CSF support. Peripheral-blood stem cells were collected using a Cobe Spectra CS 3000. Finally, patients in complete (CR) or partial response (PR) received high-dose chemotherapy with melphalan 160 mg m^−2^ plus mitoxantrone 60 mg m^−2^ or thio-tepa 600 mg m^−2^, followed by the reinfusion of the peripheral-blood stem cells. G-CSF (300 *μ*g day^−1^) starting on day +5 was administered when the number of reinfused CD34+ cells was less than 5 × 10^6^ kg^−1^.

Local surgery or radiotherapy was performed, when possible, before or after high-dose chemotherapy, on the basis of the investigators' best judgement.

### Response evaluation

Tumour response was evaluated after four IVE courses, before the myeloablative regimen and at the end of all therapies. Complete remission (CR) was defined as the absence of any detectable tumour; pertial remission (PR) was defined as a reduction in all measurable tumours by more than 50%; stable disease (SD) was defined as no tumour growth, in the absence of new lesions and reduction in any measurable tumour by less than 50%; progressive disease (PD) was defined as tumour growth by more than 25% in volume or the appearance of any new lesions.

The side effects were graded using the toxicity criteria of the National Cancer Institute.

### Molecular study

Molecular analyses were made on peripheral blood and bone marrow at the time of diagnosis, on peripheral blood alone after the induction phase, on the leukapheresis products, and during follow-up.

### Sample preparation and RNA extraction

RNA was extracted from the peripheral-blood and bone marrow samples, and isolated by TRIzol reagent (Gibco BRL) according to manufacturer's directions, and resuspended in DEPC-treated water.

### RT–PCR

A measure of 1 *μ*g of total RNA was reverse-transcribed using 200 U of Maloney murine leukaemia virus RT (SuperScript II, Gibco BRL), in a 50-*μ*l reaction volume containing 20 mM Tris-HCl, 50 mM KCl pH 8.3, 0.5 mM dNTPs, 10 mM DTT, and 300 ng of random primers. Templates and random hexamers were first incubated at 65°C for 10 min, and reverse transcription was performed at 42°C for 90′; the procedure was stopped by enzyme inactivation at 70°C for 10′.

Templates were amplified in standard conditions by a single reaction in the presence of 10 pmol of both WT.1 (5′-GCC ACC GAC AGC TGA AGG GC-3′-3′) and EWS22.3 (5′-TCC TAC AGC CAA GCT CCA AGT C-3′) primers in the following cycling conditions: 94°C for 10′, 35 cycles at 94°C for 30″, 65°C for 30″, 72°C for 60″, and a final extension at 72°C for 10′. The expected fragment lengths vary according to the translocation type from 259 to 522 bp.

All cDNAs were checked for the expression of the ubiquitously expressed *β*-actin gene.

### Statistical methods

Survival analysis was determined using the Kaplan–Meier product-limit method, by computing the number of days elapsing between the date of the start of treatment and either the date of death or last contact for overall survival or progression for progression-free survival. The differences between the curves were evaluated using the log-rank test.

## RESULTS

### Patient characteristics and results

In all, 10 consecutive patients were treated from 1997 to 2002. All patients were male and the median age was 29 years. Locally advanced disease was diagnosed in six patients, while four patients had metastatic disease.

After induction chemotherapy, five out of 10 patients (50%) achieved a PR, two out of 10 patients (20%) had SD, and three out of 10 patients (30%) progressed. Responsive patients received high-dose chemotherapy, but no CR conversion was obtained. Overall, six out of 10 patients (60%) proceeded to surgery (two patients after and four patients before HDCT); surgery was radical in three patients. The overall response rate after chemotherapy and surgery was 60% (CR 30%, PR 30%), while 40% of patients showed PD. Treatment was well tolerated and no toxic deaths were observed.

**Figure 1:**
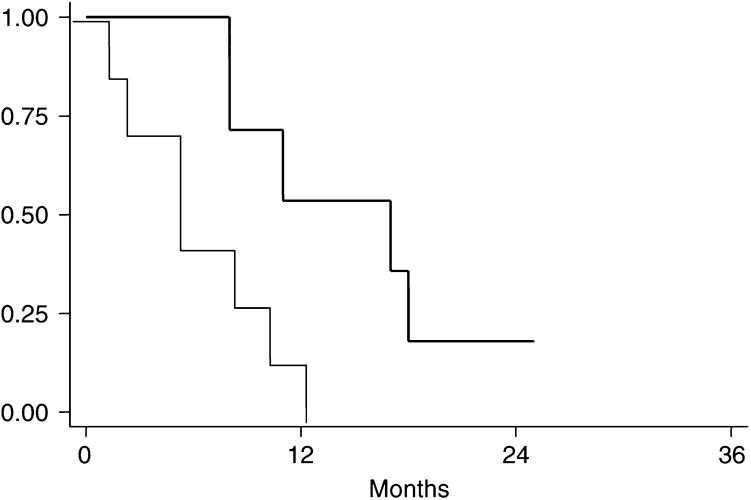
Overall survival (dotted line) and progression-free survival.

After a median follow-up of 35 months (range 14–60), the median overall survival was 14 months (range 7–25) (Figure 1

).

**Figure 2:**
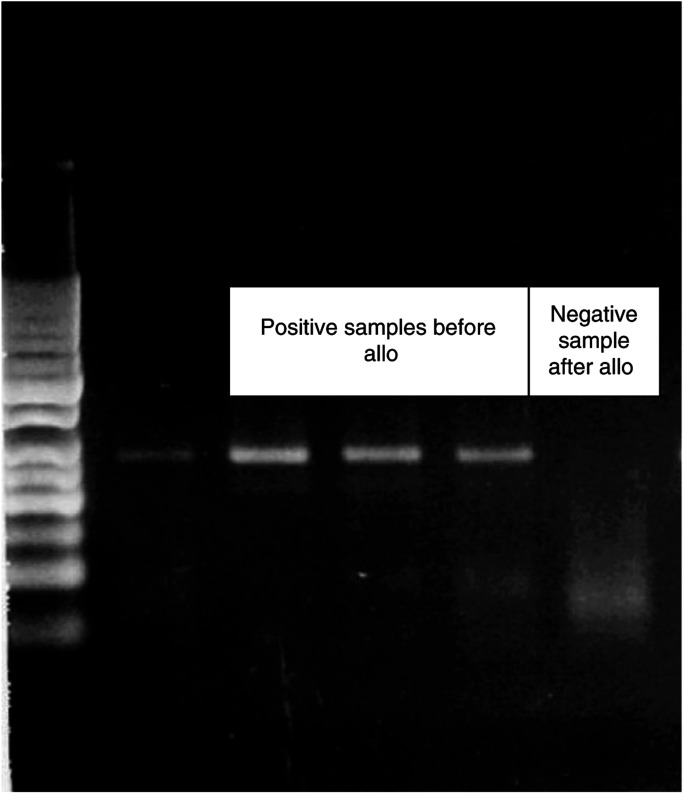
Molecular results in patients treated with allogeneic stem cell transplantation with a reduced-intensity conditioning regimen.

EWS-WT1 fusion transcript was positive in all patients at diagnosis in the histological samples, peripheral blood, and bone marrow. The detection of molecular marker was always positive in peripheral blood in all patients, when evaluated during treatment and in leukapheresis products. Only one patient submitted to allogeneic peripheral stem cell transplantation after a reduced-intensity conditioning regimen showed the disappearance of fusion transcript at the time of cyclosporine tapering and acute graft versus host disease (GvHD) development (Figure 2

).

## DISCUSSION

The results of this prospective study suggest that high-dose chemotherapy does not improve clinical results in DSRCT. Indeed, partial responders after induction chemotherapy did not show a CR conversion with high-dose chemotherapy. Furthermore, no complete responders ever obtained clearance of EWS-WT1 fusion transcript, during the different steps of treatment, and the median overall survival remained dismal (14 months).

Interesting data have been previously reported in literature by Kusher *et al*, using an intensive alkylator-based chemotherapy combined with surgery and radiotherapy, with a 3-year overall survival of 29%. The chemotherapy regimen is aggressive even if in their original report only four (CR – two, PR – two) out of 12 patients received high-dose chemotherapy with PSC support and the two PRs were not converted to CR. Two main differences were evident compared to the present series: the higher dose of alkylator and the application of radiotherapy to the whole abdomen. However, after the chemotherapy, response rate (66%) was comparable to that achieved in our series (60%) and with regard to radiotherapy (Goodman *et al*, 2002), mainly hematological and intestinal toxicity (bowel obstruction in 33%), is considerable, and radiotherapy does not seem to modify the overall results.

Considering the presence of a recurrent translocation with a new hybrid transcript with antigenic proprieties in DSRCT (Worley *et al*, 2001), we treated a patient progressing after chemotherapy and surgery with matched sibling allograft using a reduced-intensity conditioning regimen. The patient rapidly obtained a full donor chimerism and cyclosporine was tapered when tumour progression was documented. He developed acute GvHD (grade III) and extensive chronic GvHD. Molecular monitoring of the presence of hybrid transcript in peripheral blood, previously positive, was converted to negative after GvHD development (Figure 2). This phenomenon could be interpreted as a graft versus desmoplastic effect.

In conclusion, high-dose chemotherapy does not change the fate of DSRCT. Considering the low incidence of this tumour, cooperative studies should be planned. Furthermore, allogeneic stem cell transplantation should be more extensively explored in these young patients.
